# Melatonin for chronic back pain (the MOCHA trial): study protocol for a randomized, double-blind, placebo-controlled trial

**DOI:** 10.1186/s13063-025-09206-w

**Published:** 2025-11-17

**Authors:** Kübra Kilic, Henrik Bjarke Vægter, Karin Due Bruun, Werner Vach, Jan Hartvigsen, Bart Willem Koes, Preben Kidmose, Jens Søndergaard, Jonas Bloch Thorlund

**Affiliations:** 1 Pain Research Group, Pain Center, Department of Anesthesiology and Intensive Care, Odense University Hospital, Odense, Denmark; 2 Department of Clinical Research, Faculty of Health Sciences, University of Southern, Odense, Denmark; 3 Basel Academy for Quality and Research in Medicine, Basel, Switzerland; 4 Center for Muscle and Joint Health, Department of Sports Science and Clinical Biomechanics, University of Southern Denmark, Odense, Denmark; 5 Chiropractic Knowledge Hub, Odense, Denmark; 6 Research Unit for General Practice, Department of Public Health, University of Southern Denmark, Odense, Denmark; 7 Department for General Practice, Erasmus University Medical Center, Rotterdam, The Netherlands; 8 Electrical and Computer Engineering, Department of Engineering, Aarhus University, Aarhus, Denmark

**Keywords:** Pain, Chronic back pain, Insomnia, Sleep, Melatonin, Pain sensitivity, Ear-EEG

## Abstract

**Background:**

Chronic back pain remains a leading cause of disability worldwide, with high societal and healthcare costs and limited effective treatment options. More than 50% of people with chronic back pain also report insomnia symptoms. Melatonin, primarily known for its use in treating insomnia and jetlag, has shown promising effects as a pain medication in chronic non-musculoskeletal pain conditions. We aim to determine the efficacy of 6 weeks of melatonin compared with placebo in reducing average pain intensity in patients with chronic disabling back pain.

**Method:**

The Melatonin for Chronic Back Pain (MOCHA) trial is a 1:1 randomized, placebo-controlled, double-blind, superiority trial including 220 patients with chronic disabling back pain randomized to either 10 mg (given as two 5 mg tablets) melatonin daily for 6 weeks or an identically looking placebo tablet. The primary outcome is the between-group difference in change in average pain intensity during the last 7 days from baseline to 6 weeks. Secondary outcomes include insomnia severity, back pain-related disability, global perceived effect, physical and mental health, and pain sensitivity. Exploratory outcomes are physiological sleep metrics assessed with ear electroencephalography (EEG).

**Discussion:**

This trial evaluates the efficacy of melatonin, an inexpensive and widely available intervention that could potentially reduce pain and sleep problems in a population with few effective treatment options available.

Trial registration

CTIS EU-CT 2023-503530-41-00. Registered on March 4th, 2024.

ClinicalTrials.gov NCT06476392. Registered on June 20th, 2024.

## Administrative information

Note: the numbers in curly brackets in this protocol refer to SPIRIT checklist item numbers. The order of the items has been modified to group similar items (see http://www.equator-network.org/reporting-guidelines/spirit-2013-statement-defining-standard-protocol-items-for-clinical-trials/).

**Table:** 

Title {1}	Melatonin for Chronic Back Pain (The MOCHA trial): Study Protocol for a Randomized, Double-Blind, Placebo-Controlled Trial.
Trial registration {2a and 2b}.	**CTIS:** EU-CT 2023–503530-41-00 (Registered on March 4th 2024) **ClinicalTrials.gov ID: **NCT06476392 (Registered on June 20th 2024)
Protocol version {3}	Version 4.0 (26.08.2024)
Funding {4}	The study is funded by grants from (Sygesikringen “danmark”—Health Insurance Denmark) and The Region of Southern Denmark's Fund for Independent and Strategic Research.
Author details {5a}	Kübra Kilic^1,2^,Kubra.kilic@rsyd.dk Henrik Bjarke Vægter^1,2^,hbv@rsyd.dk Karin Due Bruun^1^,Karin.Due.Bruun@rsyd.dk Werner Vach^3^,werner.vach@basel-academy.ch Jan Hartvigsen^4,5^,jan.hartvigsen@health.sdu.dk Bart Willem Koes^6,7^,b.koes@erasmusmc.nl Preben Kidmose^8^,pki@eng.au.dk Jens Søndergaard^6^,JSoendergaard@health.sdu.dk Jonas Bloch Thorlund^4,6^,jthorlund@health.sdu.dk ^1^Pain Research Group, Pain Center, Department of Anesthesiology and Intensive Care, Odense University Hospital, Odense, Denmark. ^2^Department of Clinical Research, Faculty of Health Sciences, University of Southern Denmark, Odense, Denmark. ^3^Basel Academy for Quality and Research in Medicine, Basel, Switzerland. ^4^Center for Muscle and Joint Health, Department of Sports Science and Clinical Biomechanics, University of Southern Denmark, Odense, Denmark. ^5^Chiropractic Knowledge Hub, Odense, Denmark ^6^Research Unit for General Practice, Department of Public Health, University of Southern Denmark, Odense, Denmark. ^7^Department for General Practice, Erasmus University Medical Center, Rotterdam, The Netherlands. ^8^Electrical and Computer Engineering, Department of Engineering, Aarhus University, Aarhus, Denmark.
Name and contact information for the trial sponsor {5b}	Karin Due Bruun, MD, PhD. Pain Research Group, Pain Center, Department of Anesthesiology and Intensive Care, Odense University Hospital, Heden 7–9, 5000 Odense, Denmark Karin.Due.Bruun@rsyd.dk
Role of sponsor {5c}	The study is investigator initiated. Consultant Karin Due Bruun, PhD who is an independent researcher at the Pain Research Group at Odense University Hospital is sponsor and primary investigator and is involved together with the other researchers in study design, collection, management, analysis, and interpretation of data, writing of the report, or the decision to submit the report for publication. The funders do not have any part in the design, conduct or dissemination of the trial. The investigators retain authority over all these activities.

## Introduction

### Background and rationale {6a}

Chronic back pain affects more than 600,000 million people globally, and the prevalence is expected to increase in the coming decades [1, 2]. It is a leading cause of disability and is associated with enormous healthcare and social costs [1, 2]. The underlying mechanisms for chronic back pain are not fully understood, which complicates management [3]. Many patients with chronic back pain use pain medication such as paracetamol, anti-inflammatory drugs (NSAIDs), and opioids [4–6]. However, the effect of these pain medications compared with placebo is modest, with effects typically less than 10 points on a 0–100 pain scale [5–7]. In addition, the use of these analgesics is associated with an increased risk of gastrointestinal and cardiovascular side effects and a risk of dependency, tolerance, and addiction [4–6]. Consequently, the World Health Organization (WHO) does not recommend the use of these pain medications for chronic back pain, except for NSAIDs for short periods, and only for patients younger than 60 years of age [8].

Pain and insomnia often co-occur, with more than 50% of chronic back pain patients reporting insomnia symptoms [9, 10]. Research suggests that insomnia has negative effects on pain processing [9, 10], and that insomnia predicts the development and worsening of future pain. Consequently, people with insomnia report more pain, and improvement of insomnia has been associated with improvements in pain [9, 11, 12].

Melatonin is a hormone excreted by the pineal gland that regulates the circadian rhythm and sleep [13, 14]. Melatonin is also a widely available drug well known for its use in people with insomnia and jetlag [13, 15]. It has a favorable safety profile with no reported side effects of major clinical significance and is well tolerated [16, 17] with low potential for dependency and rebound effect [13]. Preliminary studies in non-musculoskeletal pain conditions such as migraines, endometriosis, and irritable bowel syndrome have shown that melatonin may have an analgesic effect [18–21] with effects possibly mediated, e.g., through direct effect on pain processing [22, 23] or indirectly through improved sleep [22]. Further, studies have shown a promising effect of melatonin for fibromyalgia pain [23–25] but there are no large randomized studies investigating the effect of melatonin on chronic back pain [21].

### Objectives {7}

The primary aim of this study is to investigate if daily treatment with melatonin 10 mg (given as two 5 mg tablets) compared with an identically appearing placebo tablet (lactose monohydrate, potato starch, mucilago gelatinae 4%, magnesium stearate, and talc) once daily before bedtime for 6 weeks is superior in reducing pain intensity in patients with chronic disabling back pain. We will also explore whether part of the effect is mediated through reduced pain sensitivity assessed with pressure algometry or improved physiological sleep assessed by an ear electroencephalography (ear-EEG) device (in a subgroup of participants).

### Trial design {8}

This study is a 1:1 randomized, placebo-controlled, double-blind, single-center parallel-group superiority trial.

## Methods: participants, interventions, and outcomes

### Study setting {9}

This study is conducted at the Pain Center, Department of Anesthesiology and Intensive Care, Odense University Hospital, Heden 7–9, 5000, Odense, Denmark. The setting is a tertiary care university hospital pain center.

### Eligibility criteria {10}

#### Inclusion criteria

Danish speaking/understanding adults aged 18 to 64 years with back pain in the area from T1 to the gluteal fold. Back pain must be present on “most days” or “every day” for the past 3 months, back pain must limit daily life or work activities on “some days,” “most days,” or “every day” during the past 3 months, and the average pain intensity over the past 7 days must be 4 on 0–10 Numeric Rating Scale (NRS) [26]. All fertile women must use safe contraception (spiral, birth control pills, contraceptive patch, contraceptive vaginal ring, or progestin injections) for 3 weeks before and 1 week after the trial. If the female participants do not use safe contraception, they must provide oral and written informed consent that they will not engage in sexual activity with males during the trial. A woman is considered non-fertile if she is sterilized, hysterectomized, bilateral oophorectomized, or is postmenopausal. A woman is considered postmenopausal when vaginal bleeding has been absent for 1 year as reported by the participant.

#### Exclusion criteria

People with known abuse of alcohol or other substances or people who use opioids. People with known malignancies within the past 6 months, known fractures within the past 4 months, lumbar radiculopathy, spinal stenosis, and inflammatory/autoimmune arthritis. People with severe psychiatric disorders, suicide and self-damage thoughts and/or psychotic symptoms. It is required to have a negative urine test for human chorionic gonadotropin (hCG) for pregnancy for fertile women before inclusion. A blood test (serum levels of alanine aminotransferase (ALAT), creatinine, glomerular filtration rate (GFR)) will ensure that there is not any kidney or liver failure before inclusion.

If a participant fulfills one of the contraindications to melatonin according to the Danish Medicines Agency’s approved product information (supplement), they will be excluded.

For the ear-EEG subgroup, participants will be excluded if the anatomy of the outer ear makes it impossible to do an ear-mold, if they have a perforation of the tympanic membrane, or an ear tube in the tympanic membrane.

### Who will take informed consent? {26a}

The investigating medical doctors (KK or KDB), or a medical doctor that has been trained by KK or KDB in the study procedure, will be responsible for the informed consent procedure. Informed consent is obtained and signed by the participant and co-signed by the investigator at visit 1 before data collection and before accessing the participant’s medical file, including the shared medication record and laboratory data.

Participants are informed that participation is voluntary, and they can withdraw consent at any time.

### Additional consent provisions for collection and use of participant data and biological specimens {26b}

The handling of data will be in accordance with Regulation 2016/679 of the European Parliament and of the Council of 27 April 2016 on the protection of natural persons with regard to the processing of personal data and on the free movement of such data (GDPR) and with the Danish Data Protection Act. In addition to consent to participate in the MOCHA main trial, consent to participate in the ear-EEG sub-study is also provided by these participants. The blood samples which are used as part of the inclusion/exclusion criteria are managed in accordance with the GDPR and the Danish Data Protection Act. None of the blood samples will be stored or sent abroad and will not be used for future research.

## Interventions

### Explanation for the choice of comparators {6b}

With lack of an established standard treatment for back pain, placebo tablets of identical appearance to the active melatonin drug is used as comparator to investigate if melatonin has a pain relieving effect.

### Intervention description {11a}

After randomization, participants receive a supply of either 10 mg (given as two 5 mg tablets) melatonin tablets or placebo tablets for the entire 6-week study period. They are instructed to take two tablets orally once daily before bedtime for 6 weeks. The melatonin test dose is based on findings from a Spanish dose–response study [24].

### Criteria for discontinuing or modifying allocated interventions {11b}

If tolerability issues related to the study medication arise during the study period, the dose will be reduced to 1 tablet (5 mg/day) or the treatment will be discontinued depending on the case. See Sect. 22 for details about adverse events and harms.

Discontinuation of the allocated intervention will also occur if the participant withdraws consent.

### Strategies to improve adherence to interventions {11c}

A weekly reminder will be sent to the participants via e-Boks, Denmark’s official and secure digital mailbox system, reminding them to take their trial medication.

before bedtime and to complete the weekly outcome questionnaire (see item 18b).

At week 3, a blinded investigator will conduct a scheduled safety phone call to assess adherence, reinforce medication compliance, and inquire about any adverse events. At visit 3 (week 6), participants will return any remaining trial medication for tablet count. Compliance will be calculated as the proportion of tablets taken relative to expected intake and documented in the eCRF. These measures provide both behavioral support and quantitative assessment of adherence.

### Relevant concomitant care permitted or prohibited during the trial {11d}

Participants are allowed to continue usual concomitant care for their back pain but are asked not to start new interventions (e.g., pain medication use, exercise therapy, and manual therapy). Opioids and other drugs that are considered contraindications with melatonin use according to the Danish Medicines Agency’s approved product information are not permitted. This will be monitored by a self-reported questionnaire and the shared electronic medication record.

### Provisions for post-trial care {30}

If participants still have a need for care for their back pain after the trial, they will be recommended to contact their general practitioner.

Participating patients are covered by the governmental patient insurance, which includes all patients in the Danish health care system.

### Outcomes {12}

#### Primary outcome

The primary outcome is the difference between groups (melatonin vs. placebo) in change in *average pain intensity* over the last 7 days from baseline to 6 weeks assessed using a 0–10 Numeric Rating Scale (NRS), with 0 indicating “no pain” and 10 indicating “worst imaginable pain” [17].

#### Secondary outcomes

For the assessment of *back pain-related disability*, the 23-item Roland Morris Disability Questionnaire (RMQ) is used [27, 28], as it is validated cross-culturally in Danish and its psychometric properties have been demonstrated to be comparable to those of the 24-item version [29, 30]. Each item is scored as yes (1 point) or no (0 points), with a total score range of 0 (no disability) to 23 (extremely severe disability).

For the assessment of *global perceived effect* (GPE) after 6 weeks, the participants will respond to the question: “How is your back pain now compared to when you entered this study,” with 5 response options (much worse, worse, almost the same/unchanged, better, and much better) [31].

*Insomnia symptoms* will be assessed by the Insomnia Severity Index (ISI), which is a 7-item questionnaire with a score ranging from 0 to 28 (0 = best; 28 = worst) [32, 33].

*Physical and mental health* will be assessed using the Patient-Reported Outcomes Measurement Information System (PROMIS-10) Global Health questionnaire version 1.2 [34]. The PROMIS-10 questionnaire comprises 10 items designed to evaluate multiple dimensions of global health. A Physical Health and Mental Health score will be calculated from 8 of the 10 questions. The first 9 questions utilize a Likert scale with 5 response options, while the last question assesses pain using a 0–10 numeric rating scale.

*Pain sensitivity:* Pressure pain thresholds (PPT) will be assessed using a handheld algometer (Somedic Sales AB, Norra Mellby, Sweden) [35, 36] with a stimulation area of 1 cm^2^ and a pressure rate of 30 kPa/s at two specific body sites: the right erector spinae muscle, 3 cm lateral to the fourth lumbar spinous process, and the left upper trapezius muscle, 10 cm horizontally from the acromion in alignment with the seventh cervical spinous process. Patients are instructed to press a button when the pressure is perceived as the first sensation of minimal pain. Two assessments with 20-s intervals between assessments are completed for each site, and the average at each site is used for analysis.

#### Exploratory outcomes (not to be reported in primary manuscript)

*Physiological sleep metrics* will be collected as an exploratory outcome and reported in secondary publications. Assessment of physiological sleep metrics will be performed using ear EEG in a subgroup of approximately 60 patients (aiming for melatonin (*n* = 30) vs. placebo (*n* = 30)). Physiological sleep will be assessed at home for 5 nights at baseline and in week 4 using an ear-EEG research solution (T&W Engineering A/S, Lynge, Denmark) that is custom-fitted for each individual patient. The 60 participants for the EEG subgroup will be included consecutively after the inclusion of the first 100 participants among those participants who are eligible for ear-EEG and consent to participate in the ear-EEG measurements. Sleep variables will be derived from the ear-EEG assessments as recommended by the American Academy of Sleep Medicine (AASM)[37] and will include the following sleep metrics:

- *Total sleep time (TST):* The duration from sleep onset to final awakening.
- *Sleep efficiency (SE):* The ratio of TST to time in bed, expressed as a percentage.
- *Sleep onset latency (SOL):* The time taken to transition from wakefulness to sleep.
- *Wake after sleep onset (WASO):* The total duration of wakefulness occurring after initial sleep onset.
- *Time in each sleep stage N1, N2, N3, and REM:* Expressed as a percentage of SPT.
- *Number of awakenings within TST:* The count of awakenings during the total sleep time.
- *Arousal index:* The number of arousals per hour.

#### Additional baseline data

At baseline, the following data will be collected: demographic data (sex, ethnicity, work status, education), health-related lifestyle data (smoking, alcohol use, and physical activity level), medical history, concomitant medicine use, and vital signs (blood pressure, heart rate, weight, height) are measured.

### Participant timeline {13}

**Fig. 1:**
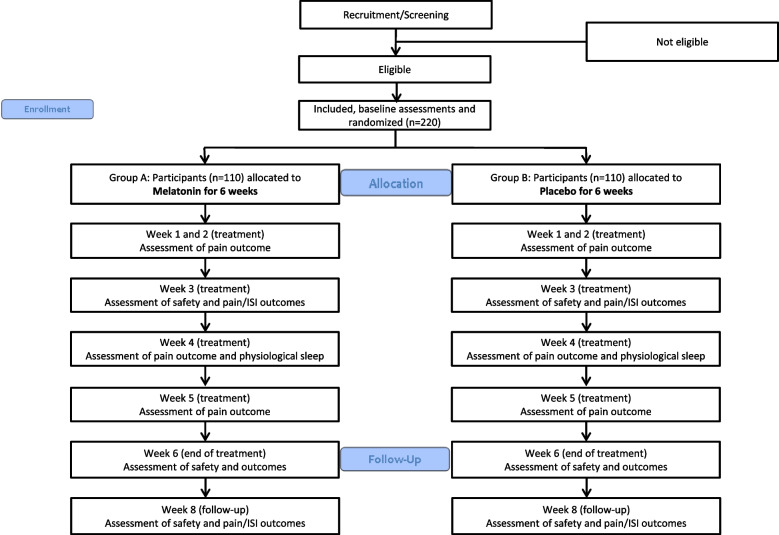
Overview of the participant flow

The participant flow is illustrated in Fig. 1 and the time schedule for enrollment, randomization, outcome assessments, and interventions is shown in Table 1.

**Table 1:** Schedule of enrollment, interventions, and outcome assessments

Week	Study period								
	**Enrollment**	**Allocation**	**Post-randomization**						**Follow-up**
	**− 4–0**	**0**	**1**	**2**	**3**	**4**	**5**	**6**	**8**
**Enrollment**									
Eligibility	x								
Informed consent	x								
Medical history	x								
Demographic data	x								
**Interventions**									
Melatonin			x	x	x	x	x	x	
Placebo			x	x	x	x	x	x	
Adherence								x	
**Safety**					x			x	x
**Outcome assessments**									
**PROMS**									
NRS pain intensity		x	x	x	x	x	x	x	x
ISI		x			x			x	x
RMQ		x						x	
PROMIS-10 GH		x						x	
GPE								x	
**Pain sensitivity**		x						x	
**Physiological sleep**									
Ear-EEG		x				x			
Adverse device effects		x						x	

*NRS* Numerical Rating Scale,* ISI* Insomnia Severity Index, *RMDQ* Roland Morris Disability Questionnaire, *GPE* global perceived effect, *PROMIS-10* GH Patient-Reported Outcomes Measurement Information System Global Health v 1.2

### Sample size {14}

Based on an unpaired t-test, aiming to detect a between-group difference of 1 point or more in the change in pain intensity and assuming a SD of 2.5, 100 participants should be included in each group to achieve a power of 80% with a significance level of 5%. This difference is similar to the effect of NSAIDs compared to placebo for acute and chronic back pain [6, 38], and the SD is similar to other randomized controlled trials investigating the effects of analgesics versus placebo in chronic back pain patients that have reported SD for change in pain intensity (e.g., [39, 40]). The intended analytical approach involves using baseline values as covariates instead of change scores, which is expected to result in a further gain in statistical power. Dropouts may decrease the statistical power; however, as a mixed model will be used for analysis, they will still contribute information. To account for any potential loss of power, the sample size is increased by 10%, i.e., we aim at a total sample size of 220 patients.

### Recruitment {15}

Participants will be recruited through advertisements (local papers and local radio), social media campaigns (LinkedIn and Facebook), and from the pain center waiting list. Recruitment via LinkedIn and Facebook is done through general posts and posts targeting special interest groups for people with chronic pain. Participants expressing interest in the study are contacted by phone for an initial screening in relation to the eligibility criteria by one of the investigators (KK). Following the telephone screening, written information material is sent to potentially eligible participants, and they are invited to a consultation at the Pain Center where the study information is repeated verbally, inclusion and exclusion criteria are assessed, and informed consent is obtained.

## Assignment of interventions: allocation

### Sequence generation {16a}

A computerized algorithm will be generated using REDCap electronic data capture tools to prepare a randomization list of 220 sequential numbers (110 in each group) allocating the participants to the melatonin- or placebo group (1:1 allocation ratio). The randomization will be in permuted blocks of 2–6 individuals. No stratification factors will be used.

### Concealment mechanism {16b}

The randomization list will be prepared by a data manager, who is not otherwise involved in the study. The allocation sequence is concealed in a password-protected computer file, which only the data manager has access to. The randomization list is sent to the pharmacy, which will label the medicine with blinding codes according to the list. The trial medicine will be delivered to the trial site together with individual code-envelopes for every blinding code, and the trial site is responsible for the reception control**.** The coded envelopes will be stored behind double lock with 24/7 accessibility at the department of Anesthesiology and Intensive Care at Odense University Hospital (OUH).

### Implementation {16c}

The participants will be enrolled by the investigator KK, or the primary investigator and sponsor KDB, or a medical doctor that has been trained in the study protocol. After enrollment, the investigator concerned will assign the participants a computer-generated sequential number that will randomly allocate them to either the intervention or the control group.

The allocation sequence is generated by an independent data manager using a computerized algorithm, aligning with details from Sect. 16a.

## Assignment of interventions: Blinding

### Who will be blinded {17a}

The study is double-blinded as all participants, investigators, outcome assessors, and statistical analysts will be blinded to treatment allocation. Placebo tablets and the active medicine, melatonin, will appear completely identical and be blinded in identical cans labeled with blinding codes.

### Procedure for unblinding if needed {17b}

Unblinding will occur after the analysis of the primary outcome. During the study period, unblinding of a single participant will only occur in the case of a medical emergency and if the Sponsor/PI (KDB) or the investigator finds it necessary to ensure the safety of the participant. Unblinding occurs by breaking the code-envelope related to the subject’s blinding code. In the case a code-envelope is opened, it will be stored in the trial master file.

## Data collection and management

### Plans for assessment and collection of outcomes {18a}

Figure 1 and Table 1 describe the plan for screening, enrollment assessments and interventions and collection of the outcomes from baseline to the end of trial after 6 weeks. The safety assessment at week 3 (midway safety assessment) and week 8 (end of follow-up assessment) will be performed via telephone. Between trial start and end-of-trial, participants will be asked to respond to questionnaires related to their average pain intensity during the last week once a week for 6 weeks.

The exploratory outcome (physiological sleep metrics) is assessed in a subgroup of 60 participants who will have an extra visit before the start of the trial (Table 1). During this visit, ear-EEG related procedures will be made: molding of customized ear-EEG and initial test of the device, following monitoring of the sleep with the ear-EEG.

Data are collected in the electronic Case Report Forms (eCRFs), and data collection templates can be made available upon request.

### Plans to promote participant retention and complete follow-up {18b}

Participants will receive weekly electronic reminders via e-Boks, Denmark’s official secure digital communication platform, to complete outcome questionnaires. Non-responders will receive up to three additional daily reminders during the same week. If unanswered, the investigator (KK) will follow up via SMS and telephone. In week 3, participants will be contacted by an investigator (KK) blinded to treatment allocation to encourage questionnaire completion and adherence to trial medication. Participants withdrawing from the study will be encouraged to complete all planned questionnaires.

### Data management {19}

Participants will respond to questionnaires via the REDCap electronic data capture tools that enter data directly into the electronic Case Report File (eCFR). Investigator-collected data will be entered directly into the eCFR during the visits. Range checks for data values will be used to improve the data quality in the eCFR. The ear-EEG data are saved in a secured and logged SharePoint. In the analyzing process, all the data will be transferred to a statistical program. The data will be anonymized and stored 25 years after the end of the trial. The Trial Master File (TMF) is saved in a safe and password-protected SharePoint.

### Confidentiality {27}

All personal data related to potential or enrolled participants will be collected in a secure and logged database, SharePoint, or behind double lock if it is in paper format. Data exports will only include a study-specific identifier. All these precautions will be made to protect the confidentiality before, during, and after the trial.

Five years after study termination, all data will be anonymized. In accordance with Regulation (EU) No. 536/2014, data from clinical trials registered in CTIS (Clinical Trials Information System) must be retained for a minimum of 25 years following study completion. Therefore, the data deletion date has been adjusted accordingly. All collected data will be stored in a secure system for 25 years, in compliance with applicable data protection regulations.

### Plans for collection, laboratory evaluation and storage of biological specimens for genetic or molecular analysis in this trial/future use {33}

Blood samples will be collected and analyzed to assess the liver and kidney function before inclusion at baseline (no blood samples will be stored for research purposes). The Department of Clinical Biochemistry at Odense University Hospital will be responsible for the blood sample analyses and for the destruction of the blood samples after the analysis. The samples are managed in accordance with the GDPR and the Danish Data Protection Act.

## Statistical methods

### Statistical methods for primary and secondary outcomes {20a}

The main analyses will be based on the intention-to-treat (ITT) population. Estimation of the treatment effect on the primary and secondary outcomes will be based on mixed linear models with time (baseline and any follow-up time point), treatment group (melatonin or placebo), and the interaction between time and treatment arm, as fixed effects and intercept and slope as patient-specific random effects. The error variance will be allowed to vary over time and across the two groups, except at baseline. The variance-covariance matrix of the random effects will be allowed to vary across the two groups. Interactions will be parametrized as time-dependent treatment effects such that the treatment effect at week 6 corresponds directly with the efficacy parameter of interest. The treatment effect at baseline will be set to 0 to reflect randomization. The treatment effect will be expressed as the gain in reduction observed in the Melatonin group, i.e., a positive number expresses a favoring of Melatonin.

For the primary outcome, the follow-up time points 1, 2, 3, 4, 5, and 6 weeks will be used. For the secondary outcomes, the follow-up time points 3 and 6 weeks will be used. Fitting of the mixed models will be based on the restricted maximum likelihood technique. In case of convergence problems, the model will be simplified by additional restrictions on the variance parameters with respect to equality over time and between the two groups.

In two separate responder analyses, a responder is defined as a patient who reports more than 30% and 50% decrease in pain intensity after 6 weeks, respectively, and differences between treatment groups will be expressed by adjusted odds ratios. These will be based on a logistic regression model including the baseline pain intensity value as a covariate.

Table 2 describes the intended trial interpretation for all possible effect estimates and 95% CI scenarios. A between-group difference of 1 point in the primary outcome is considered clinically relevant. Statistical significance is evaluated for the primary outcome and all secondary outcomes at the 5% level. No adjustment for multiplicity is performed.

**Table 2:** Trial interpretation for different estimate and 95% CI scenarios

**Scenario**	**Estimate**	**Lower bound CI**	**Upper bound CI**	
1	≥ 1	≥ 1		We have demonstrated an intervention effect of clinically relevant magnitude
2	≥ 1	≥ 0, ≤ 1		We have demonstrated an intervention effect and obtained a positive intervention effect estimate of clinically relevant magnitude
3	≥ 1	≤ 0		We obtained a positive intervention effect estimate of clinically relevant magnitude, but we could not demonstrate an intervention effect
4	≥ 0, ≤ 1	≥ 0	≥ 1	We have demonstrated an intervention effect and obtained a positive intervention effect estimate
5	≥ 0, ≤ 1	≥ 0	≤ 1	We have demonstrated an intervention effect but also the absence of an intervention effect of clinically relevant magnitude
6	≥ 0, ≤ 1	≤ 0	≥ 1	We obtained a positive intervention effect estimate, but we could not demonstrate an intervention effect
7	≥ 0, ≤ 1	≤ 0	≤ 1	We obtained a positive effect estimate but also demonstrated the absence of an intervention effect of clinically relevant magnitude
8	≤ 0		≥ 1	We obtained a negative intervention effect estimate, but we could not exclude that the intervention effect is of clinically relevant magnitude
9	≤ 0		≥ 0, ≤ 1	We obtained a negative intervention effect estimate and demonstrated the absence of an intervention effect of clinically relevant magnitude
10	≤ 0		≤ 0	We have demonstrated the absence of a positive intervention effect

### Interim analyses {21b}

No interim analysis will be conducted.

### Methods for additional analyses (e.g., subgroup analyses) {20b}

Additional statistical analyses beyond those predefined in this protocol, including mediation analysis and effect modifiers, are planned. A detailed SAP will be developed and documented prior to the inclusion of the last patient in the study. The SAP will in particular include:

- A list of candidate variables to be analyzed for mediation and effect modification.
- A strategy to perform these analyses.

### Methods in analysis to handle protocol non-adherence and any statistical methods to handle missing data {20c}

The use of a linear mixed model for all longitudinal outcome measurements, including baseline, allows to include all randomized patients in the primary analysis, independent of the missing patterns observed. Consequently, the intention-to-treat (ITT) principle can be applied, assuming that missing outcome data satisfies the missing at random assumption.

Sensitivity analyses with respect to the missing at random assumption will be conducted. These analyses will be based on multiple imputation and follow two different approaches. First, we will consider a tipping approach adding to all generated outcome values a constant value $\updelta$, mimicking the expectation that missing outcomes values are in reality higher than those we would expect under the missing at random assumption [41, 42]. The second approach focus on patients in the melatonin group, who stop participation in the trial, i.e., for whom both the outcome values as well as information on the medication is missing after the finally observed outcome. In these patients, the further increments are generated using the distribution of the increments observed in the placebo group in patients with similar values until drop out [43].

At the end of the intervention phase, empty medicine-cans will be returned, and any non-ingested medicine will be counted by a trained employee at the trial site. Participants will be asked if they lost any of the tablets. Both the number of returned tablets and the number of lost tablets will be recorded. It is required for the participants to have an adherence to the active medication and placebo of at least 80% during the study period to be defined as “compliant.” A per-protocol analysis will be performed including only compliers.

### Plans to give access to the full protocol, participant level-data and statistical code {31c}

The CTIS protocol and a detailed statistical analysis plan (SAP) will be made publicly available at www.CclinicalTtrials.gov with the identifier: NCT06476392.

## Oversight and monitoring

### Composition of the coordinating center and trial steering committee {5d}

The study is coordinated by the sponsor and principal investigator (KDB) KK, JBT, and HBV (trial steering committee). The committee meets 4 times a year to discuss the progression of the study.

### Composition of the data monitoring committee, its role and reporting structure {21a}

The data monitoring committee (DMC) meets every 2 weeks. The DMC is composed of KK, JBT, and HBV. The DMC reviews the recruitment progress, data adherence rate, and the quality of the data (e.g., ear EEG).

### Adverse event reporting and harms {22}

Adverse events (AEs) and adverse reactions (ARs) are documented at every visit. Participants will complete a questionnaire regarding known side effects and will also be interviewed by the investigator about any adverse events that occur throughout the trial. The severity of harms will be assessed using the Common Terminology Criteria for Adverse Events (CTCAE) version 5.0. To determine whether an AE is related to the trial medication, the investigator will refer to the Summary of Product Characteristics (SmPC) for Melatonin. All recorded AEs and ARs will be described in detail and documented in the electronic Case Report Form (eCRF).

A serious adverse event (SAE) is defined as any unexpected medical occurrence or effect that, at any dose, results in death, poses a life-threatening risk, necessitates hospitalization or extends an existing hospital stay, causes persistent or significant disability or incapacity, or leads to a congenital anomaly or birth defect. The investigator is required to report all SAEs to the sponsor within 24 h. The causality of an SAE will be determined based on the CT-3 guidelines for collecting, verifying, and presenting adverse event/reaction reports from clinical trials involving medicinal products for human use.

If a serious adverse reaction (SAR) is deemed unexpected according to the SmPC, the sponsor must unblind the affected participant before reporting the incident to the Danish Medicines Agency, while the PI remains unblinded. In accordance with Sect. 89(2)(i) of the Danish Medicines Act, the sponsor must promptly inform the Danish Medicines Agency of any suspected unexpected serious adverse reactions (SUSARs) that occur during the trial.

In case of harms, the participants are covered by the governmental patient insurance, which includes all patients in the Danish health care system. This insurance will also cover in case of harm from the trial participation.

### Frequency and plans for auditing trial conduct {23}

The Good Clinical Practice (GCP) Unit at Odense University Hospital (https://gcp-enhed.dk/english/) monitors the trial.

The GCP unit will monitor 100% of all data on 10% of the included patients and all critical data (i.e., consent, inclusion/exclusion criteria, primary outcome, and SAE) on 100% of included patients to ensure compliance with Good Clinical Practice.

### Plans for communicating important protocol amendments to relevant parties (e.g., trial participants, ethical committees) {25}

In case of any important protocol amendments, a description of each amendment and its rationale will be reported in the Clinical Trials Information System (CTIS), where the Danish Medicines Agency and the local ethical committee will be informed. Furthermore, the monitors of the trial, the participating investigators, trial participants, and relevant trial registries such as www.ClinicalTrial.gov will also be informed in case of important protocol amendments as described above.

### Dissemination plans {31a}

The protocol and study results will be published in international peer-reviewed journals. All types of results: positive, negative, and inconclusive, will be published. The study results will be disseminated via internet media, social media, written media, news media, patient’s organizations and associations. Oral presentation and poster presentation about the study results will as well be held. The participants will be informed about the study results after the primary publication.

## Discussion

Chronic back pain is a leading cause of disability worldwide, contributing to significant healthcare costs and lost productivity. This is a study protocol for an RCT that will investigate the efficacy of melatonin on pain intensity, pain sensitivity, and physiological sleep, which could potentially be a safe and readily available treatment option to address pain and sleep for patients with chronic disabling back pain. This will be the first high-quality trial of melatonin with an adequate sample size to assess if melatonin may provide clinically important improvements in pain among patients with chronic disabling back pain. Furthermore, this randomized, placebo-controlled trial aims to generate robust evidence by minimizing the risk of bias through the blinding of participants, investigators, outcome assessors, and statistical analysts. Lastly, transparency in methodology and outcome measure definitions will be ensured through public access to the trial protocol and prior registration on ClinicalTrials.gov. If melatonin is found to be effective in relieving pain in chronic back pain patients, it could provide a novel, non-invasive treatment option that may also improve sleep, addressing two common and burdensome conditions simultaneously. This can potentially decrease the need for multiple medications and reduce the frequency of medical visits, especially at the general practitioners. Additionally, improving the quality of life for individuals with chronic back pain can enhance overall societal well-being and productivity.

The trial intervention period is 6 weeks, which was decided based on previous research [44, 45]. We consider this duration long enough to assess the analgesic effect of melatonin and potentially improve sleep quality.

## Trial status

Protocol version 4.0. Date: 26.08.2024.

Approval from the authorities: 14.03.2024.

Start of inclusion: 11.12.2024.

Expected end of inclusion: 31.08.2026.

Expected end of follow-up: 01.11.2026.

## Data Availability

The trial is investigator initiatedinvestigator-initiated and only the investigators will have access to the dataset.

## Abbreviations

AE: Adverse events
ALAT: Alanine aminotransefase
AR: Adverse reaction
E-CRF: Electronic case report form
EEG: Electroencephalography
GCP: Good Clinical Practice
GFR: Glomerular filtration rate
GPE: Global perceived effect
hCG: Human chorionic gonadotropin
IrOx: Iridium oxide
ISI: Insomnia Severity Index
N1: Non-rapid eye movement stage 1
N2: Non-rapid eye movement stage 2
N3: Non-rapid eye movement stage 3
NSAID: Non-steroidal anti-inflammatory drug
OPEN: Odense Patient data Explorative Network
PPT: Pain pressure threshold
PROM: Patient-reported outcome measure
PROMIS-10: Patient-Reported Outcomes Measurement Information System
REM: Rapid eye movement
RMQ: Roland Morris Disability Questionnaire
SAE: Serious adverse event
SAP: Statistical analysis plan
SAR: Serious adverse reaction
SE: Sleep efficiency
SmPC: Summary of Product Characteristics
SOL: Sleep onset latency
SPT: Sleep period time
SUSAR: Serious unexpected suspected adverse reactions
TST: Time from sleep onset until final awakening
WASO: Wake after sleep onset
WHO: World Health Organization
